# Diversity of *Streptococcus mutans* serotypes isolated from dental plaque of Brazilian patients with a history of acute myocardial infarction

**DOI:** 10.1590/1678-7765-2025-0077

**Published:** 2026-02-02

**Authors:** Francisco Ruliglésio Rocha, Wanessa Fernandes Matias Regis, Rochelle Pinheiro Ribeiro, João Eudes Teixeira Pinho, Tiago Lima Sampaio, Raquel Carvalho Montenegro, Ana Paula Negreiros Nunes Alves, Lidiany Karla Azevedo Rodrigues

**Affiliations:** 1 Universidade Federal do Ceará Programa de Pós-Graduação em Microbiologia Médica Departamento de Patologia e Medicina Legal Fortaleza CE Brasil Universidade Federal do Ceará, Programa de Pós-Graduação em Microbiologia Médica, Departamento de Patologia e Medicina Legal, Fortaleza, CE, Brasil; 2 Universidade Federal do Ceará Faculdade de Farmácia Departamento de Odontologia Restauradora Fortaleza CE Brasil Universidade Federal do Ceará, Faculdade de Farmácia, Odontologia e Enfermagem, Programa de Pós-Graduação em Odontologia, Departamento de Odontologia Restauradora, Fortaleza, CE, Brasil.; 3 Hospital Universitário Walter Cantídio Fortaleza CE Brasil Hospital Universitário Walter Cantídio, Fortaleza, CE, Brasil.; 4 Universidade Federal do Ceará Faculdade de Farmácia Departamento de Análises Clínicas e Toxicológicas Fortaleza CE Brasil Universidade Federal do Ceará, Faculdade de Farmácia, Odontologia e Enfermagem, Departamento de Análises Clínicas e Toxicológicas, Fortaleza, CE, Brasil.; 5 Universidade Federal do Ceará Faculdade de Medicina Departamento de Fisiologia e Farmacologia Fortaleza CE Brasil Universidade Federal do Ceará, Faculdade de Medicina, Departamento de Fisiologia e Farmacologia, Fortaleza, CE, Brasil.

**Keywords:** Streptococcus mutans, Dental caries, Cardiovascular diseases, Serotyping, Collagen-binding genes

## Abstract

**Background:**

Streptococcus mutans serotypes (c, e, f, k) are linked to dental caries, with less common serotypes (e, f, k) and collagen-binding genes (CBG: cnm and cbm) suggested to be associated with cardiovascular diseases.

**Objective:**

This study investigated the presence of *S. mutans* serotypes and collagen-binding genes (CBGs) in dental plaque and their possible association with acute myocardial infarction (AMI) in adults.

**Methodology:**

A total of 31 infarcted and 17 non-infarcted patients underwent oral and blood examinations. DNA from plaque samples was analyzed using PCR to identify *S. mutans* serotypes and cnm/cbm genes. *S. mutans* was detected in 22.6% (7/31) of infarcted patients and 11.8% (2/17) of non-infarcted patients.

**Results:**

Serotype c was the most prevalent in infarcted patients (57.1%, 4/7), followed by e (42.9%, 3/7) and k (14.3%, 1/7); serotype f was not detected. Only serotype c was found in non-infarcted patients. Less common serotypes (e, k) and co-occurring serotypes (c, k) were exclusive to infarcted patients. No CBGs were detected in any serotypes. No association was found between *S. mutans* and dental caries in either group. Patients with cholesterol levels <190 mg/dL and no *S. mutans* had a 47.4% lower risk of infarction.

**Conclusion:**

Although *S. mutans*, including less common serotypes, was more prevalent in infarcted patients, no significant association with AMI was observed. Further research is needed to elucidate the role of *S. mutans* in cardiovascular diseases.

## Introduction

*Streptococcus mutans* (*S. mutan*s) is a bacterium strongly associated with dental caries.^1^ Beyond its well-known cariogenic potential, this bacterium has also been detected in atheromatous and atherosclerotic plaques, in both diseased and healthy heart valves, and calcified aortic stenosis, as reported in the scientific literature.^2,3^ These findings suggest a potential role of *S. mutans* in the pathogenesis of atherosclerosis and, consequently, in acute myocardial infarction (AMI).^4-6^

*S. mutans* is classified into four serotypes (*c, e*, *f, k*) based on the rhamnose-glucose polysaccharide structure of its cell wall.^5,7^ Serotype *c* is the most prevalent, found in 75–80% of oral strains, followed by serotype *e* (20%), *f* (5%), and *k* (2–5%).^5,7^ While serotypes *f* and *k* are rare in healthy individuals, they are more commonly found in those with cardiovascular diseases or infective endocarditis.^7^ The collagen-binding proteins Cbm and Cnm, encoded by the *cnm* and *cbm* genes, enhance *S. mutans* adhesion to endothelial cells and heart valves,^8-10^ increasing its ability to invade and colonize collagen-rich tissues such as dental pulp and root canals.^9,11^

Strains positive for the Cnm and Cbm proteins demonstrate enhanced invasion and survival potential within the cytoplasm of human coronary artery endothelial cells.^11,12^ In animal models, the *S. mutans* OMZ175 strain, which carries the *cnm* gene, accelerates the inflammatory response and the formation of atherosclerotic plaques, a primary cause of AMI.^4,13^ The prevalence of the *cnm* and *cbm* genes in oral strains from healthy individuals has been estimated at approximately 10% and 2%, respectively,^10,14^ with these genes being more frequently identified in the less common serotypes *e, f,* and *k*.^7,10,15^

Additionally, previous studies have shown that bacterial extracellular DNA (eDNA) release mechanisms contribute to *S. mutans* biofilm formation on damaged heart valves, potentially leading to infective endocarditis.^16^ Bloodstream isolates of *S. mutans* serotypes *c* and *e* exhibit increased resistance to complement-mediated immune responses compared to oral isolates.^17^ This suggests that specific *S. mutans* strains may participate in cardiovascular tissue infection, potentially increasing the risk of AMI in individuals carrying these strains.

Clinical studies have suggested a link between *S. mutans* strains carrying the *cnm* and/or *cbm* genes and a higher risk, severity, and recurrence of caries.^14,15,18,19^ Considering that the presence of *S. mutans* serotypes and their *cnm* and *cbm* genes may be associated with an elevated risk of both caries and cardiovascular diseases, studies conducted across diverse populations and countries have explored the strength of these relationships.^8,10,11,15,19,20^

Although numerous studies have examined *S. mutans* serotypes and the presence of the *cnm* and *cbm* genes, few have specifically analyzed their distribution in cardiac patients using both dental plaque samples and bacterial isolates. Even fewer have compared these results with those from healthy control groups. This study aims to investigate the presence of *S. mutans*, its serotypes (*c, e*, *f,* and *k*), and the *cnm* and *cbm* genes in dental plaque samples collected from adults with a history of AMI, as well as from volunteers without any history of heart diseases.

## Methodology

### Subjects and ethical aspects

Oral biofilm samples were collected from 31 adults with a history of AMI due to atherosclerotic coronary artery disease before the age of 50 (39.6 ± 5.2 years; 30–49 years), all of whom were followed at the Cardiology Outpatient Clinic of the Hospital de Messejana Dr. Carlos Alberto Studart Gomes in Fortaleza, Ceará State, Brazil. Information on comorbidities and sociodemographic aspects were collected during anamnesis and review of medical records. This study was approved by the Research Ethics Committee of the Federal University of Ceará (No. 29466020.1.0000.5054). The control group (non-infarcted patients) was matched to the infarcted group by age and sex, and comprised individuals without a history of heart diseases. Inclusion criteria for the control group were: no previous diagnosis of AMI, absence of acute cardiovascular symptoms in the preceding 12 months, and provision of written informed consent. Exclusion criteria included recent dental treatment within the past three months, current use of antibiotics, and the presence of autoimmune diseases, cancer, or any condition compromising the immune system. All individuals, both infarcted and non-infarcted, were recruited from the same hospital.

### Biochemical analysis and dental plaque collection

Venous blood samples were collected in tubes containing separation gel and centrifuged at 5000 rpm for 5 minutes to obtain serum. Spectrophotometric analyses were then performed using enzymatic colorimetric methods to determine the lipid profile, including total cholesterol, triglycerides, and high-density lipoprotein-associated cholesterol.^21^ Low-density lipoprotein-associated cholesterol was calculated using the Friedewald equation.^22^ Fasting glucose was also determined according to the Guidelines of the Brazilian Society of Diabetes.^23^

The presence and severity of caries lesions were assessed using the International Caries Detection and Assessment System (ICDAS).^24^ ICDAS was applied by a previously calibrated examiner (JETPF), whose training and calibration were achieved by means of a combination of methods, including the use of original case photographs, patient examinations (not specified), and the official online training program provided for the ICDAS index. The formal calibration process involved eight examiners (including JETPF) and was conducted during the clinical examination of 60 children from a municipal public school.

After ICDAS application, dental plaque samples were collected from the surfaces of permanent teeth of all study participants by the dental surgeon (JETPF). Participants were instructed not to brush their teeth for 24 hours prior to the plaque collection appointment. Plaque was collected from all surfaces of the permanent teeth in both partially and fully dentate participants, specifically targeting sites with visible biofilm accumulation. Samples were collected using Gracey curettes (Trinity^®^, São Paulo, SP, Brazil) and stored in RNA stabilization solution (RNAlater™ Ambion Inc., Austin, TX, USA) at −80°C.

### Collection of *S. mutans* isolates from dental plaque

After collection, dental plaque samples were suspended in sterile phosphate-buffered saline (PBS) and vigorously vortexed for 60 seconds to disrupt the biofilm structure and facilitate the release of bacteria embedded within the extracellular polysaccharide matrix.

Samples were aseptically thawed and plated on Mitis Salivarius Agar (Difco Laboratories, Detroit, MI, USA) containing bacitracin (0.2 U/mL; Sigma-Aldrich Co., St Louis, MO, USA), potassium tellurite (0.001%; Sigma-Aldrich Co., St Louis, MO, USA), and 20% (w/v) sucrose.^25^ Plates were incubated at 37°C in 5% CO_2_ for 48 h. Subsequently, 5–15 colonies with rough morphology suggestive of *S. mutans* were individually cultured in Brain Heart Infusion broth (Acumedia Laboratories, Lansing, MI, USA) at 37°C and 5% CO_2_ for 18 h.^26,27^ An 200-µL aliquot of each culture was then stored at −80°C until DNA extraction.

### Genomic DNA extraction

Bacterial stocks were reactivated in Brain Heart Infusion broth at 37°C and 5% CO_2_ for 18 hours. After, 50 µL of each culture was transferred to a microtube containing 1 mL of ultrapure water (Invitrogen™ Life Technologies, Carlsbad, CA, USA), homogenized by vortexing, and centrifuged at 12,000 rpm for 3 min (Eppendorf microcentrifuge, model 5415R, Hamburg, Germany). Genomic DNA was extracted from the bacterial pellet using a DNA purification kit (InstaGene™ purification matrix, Bio-Rad Laboratories, Hercules, CA, USA), following the manufacturer's instructions.^28^ The same extraction procedure was applied to dental plaque samples. DNA from each of the two sample types was stored at −20°C until PCR analysis.

### Identification of *S. mutans*, determination of serotypes, and detection of *cnm* and *cbm* genes

DNA samples from plaque and bacterial isolates were analyzed in duplicate to identify *S. mutans*, serotyping, and detection of *cnm* and *cbm* genes. PCR was performed using the Platinum^®^ PCR SuperMix Kit (Invitrogen™ Life Technologies, Carlsbad, CA, USA), specific primer pairs for *S. mutans*, serotypes (*c, e*, *f*, and *k*), *cnm* and *cbm* genes (Table 1), and template DNA, following the manufacturer's instructions. PCRs were performed in a thermocycler (Bio-Rad 184-5096; CFX96TM Touch system, Hercules, CA, USA). For *S. mutans* identification, DNA samples from both dental plaque and bacterial isolates were subjected to the following cycling conditions: 95°C for 4 min (hot start to activate Taq polymerase), followed by 30 cycles at 95°C for 1 min, 55°C for 1 min, and 72°C for 1 min, with a final extension at 72°C for 10 min.^29, 30^ A DNA sample of *S. mutans* UA159 was used as a positive control.

**Table 1:** Summary of primers used in this study.

Target	Primer	Sequence (5’-3’)	bp	Reference
*S. mutans*	gtf B-F	5′-ACTACACTTTCGGGTGGCTTGG-3	517	30
	gtf B-R	5′-CAGTATAAGCGCCAGTTTCATC-3		
Serotype c	SC-F	5'- CGGAGTGCTTTTTACAAGTGCTGG −3'	727	31
	SC-R	5'- AACCACGGCCAGCAAACCCTTTAT −3'		
Serotype e	SE-F	5'- CCTGCTTTTCAAGTACCTTTCGCC −3'	517	31
	SE-R	5'- CTGCTTGCCAAGCCCTACTAGAAA −3'		
Serotype f	SF-F	5'- CCCACAATTGGCTTCAAGAGGAGA −3'	316	31
	SF-R	5'- TGCGAAACCATAAGCATAGCGAGG −3'		
Serotype k	CEFK-F	5'- ATTCCCGCCGTTGGACCATTC C −3'	294	32
	K-R	5'- CCAATGTGATTCATCCCATCAC −3'		
cnm	cnm-DF	5'- TGGAGGTTCAGGGCAAGTATGTTGGTGATT −3'	579	10
	cnm-DR	5'- GTCTTTTGATCAGGATTGTCAACTTTAGTC −3'		
cbm	cbm-EF	5'- AGCTGAAGTTAGTGTTGTAAAACCTGCTTC −3'	393	10
	cbm-ER	5'- TAGGATCATCAACCTTAGTCAAGTACACGA −3'		

For serotypes, multiplex PCR was performed to detect serotypes *c, e*, and *f*, using the following cycling parameters: 96°C for 2 min, followed by 25 cycles of 96°C for 15 s, 61°C for 30 s, and 72°C for 1 min.^31^ To detect serotype *k*, a singleplex PCR was conducted with the following cycling parameters: 95°C for 4 min, followed by 30 cycles of 95°C for 30 s, 60°C for 30 s, and 72°C for 30 s, with a final extension at 72°C for 7 min.^32^

Multiplex PCR was performed to detect the presence of *cnm* and *cbm* genes in DNA samples containing *S. mutans* under the following conditions: 95°C for 4 min and then 30 cycles at 94°C for 30 s, 60°C for 30 s, and 72°C for 30 s, with a final extension at 72°C for 7 min.^10^ DNA samples of *S. mutans* UA159 (serotype *c*), *S. mutans* NN2002 (serotype *e*), *S. mutans* OMZ-175 (serotype *f*), and *S. mutans* YT1 (serotype *k*) were used as positive controls. DNA samples from *S. mutans* OMZ-175 (*cnm*+) and *S. mutans* YT1 (*cbm*+) were used as positive controls.

For all reactions, PCR products were analyzed by 1.5% agarose gel electrophoresis. A 100-base-pair molecular weight marker (Invitrogen™ Life Technologies, Carlsbad, CA, USA) was applied to the gel to estimate the DNA fragments obtained. Gels were stained with SYBR^®^ Safe (Invitrogen™ Life Technologies, Carlsbad, CA, USA) and photographed (Canon Powershot A640, Canon, USA) under ultraviolet light (LTA/LTB GE, Loccus Biotecnologia, São Paulo, Brazil).

### Statistical analysis

Data were tabulated in Microsoft Excel and exported to the Statistical Package for the Social Sciences (SPSS) version 20.0 for Windows. Descriptive analysis included calculating means, standard deviations, and minimum and maximum values for quantitative variables, as well as absolute and percentage frequencies for qualitative variables. Pearson's chi-square test was applied to investigate associations between independent categorical variables. Analysis of covariance (ANCOVA) was used to investigate the effect of cholesterol on age at infarction, adjusted for the age of infarcted patients at the time of dental plaque sample collection. Binary logistic regression was used to predict whether the presence of *S. mutans* and cholesterol levels were associated with infarction. A significance level of p<0.05 was used for all statistical tests.

## Results

This study included 31 patients with a history of myocardial infarction. The mean age at the time of infarction was 39.6±5.2 years (range: 30–49 years). The age of infarcted and non-infarcted patients at the time of sample collection was 46.9 ± 6.9 years (range: 30–63 years) and 45.5±8.9 (range: 28–63 years), respectively (Table 2).

**Table 2:** General characteristics of analyzed individuals.

**Variable**	**MI Group (n=31)**	**Control Group (n=17)**
**Gender**		
Male	22 (71.0%)	11 (64.7%)
Female	9 (29.0%)	6 (35.3%)
**Age**		
Age at sample collection	46.9±6.9 (30-63 years)^1^	45.5±8.9 (28-63 years)
Age at MI event	39.6±5.2 (30-49 years)^1^	-
**Risk Factors**		
Hypertension	3 (9.7%)	-
Smoking	8 (25.8%)	-
**Oral Health Parameters (ICDAS)**		
Missing teeth	8,3±5.8 (0-29 teeth)^1^	5.4±4.5 (0-17 teeth)^1^
Sound teeth (ICDAS 0)	20.0±6.4 (1-31 teeth)^1^	24.8±5.8 (11-32 teeth)^1^
Teeth with enamel caries (ICDAS 1,2 e 3)	2.5±2.3 (0-7 teeth)^1^	1.2±1.4 (0-4 teeth)^1^
Teeth with dentin caries (ICDAS 4,5 e 6)	1.1±1.8 (0-6 teeth)^1^	0.6±1.4 (0-5 teeth)^1^

1 Quantitative data: Expressed as mean±SD (range). MI: Myocardial infarction. ICDAS: International Caries Detection and Assessment System.

In the infarcted group, polymerase chain reaction (PCR) detected *Streptococcus mutans* in five plaque samples (samples 09, 13, 16, 26, and 31), representing 16.1% (5/31). Subsequent DNA analysis of 205 bacterial isolates (5–15 colonies per individual) identified two additional infarcted patients (samples 02 and 24) positive for *S. mutans*, increasing the total prevalence to 22.6% (7/31). In the control group, PCR identified *S. mutans* in two plaque samples (samples 06 and 13), representing 11.8% (2/17). DNA analysis of 95 bacterial isolates (5–15 colonies per individual) confirmed the presence of five *S. mutans* isolates in sample 06 (Table 3).

**Table 3:** Identification of *S.mutans*, serotypes, and *cnm* and *cbm* genes in all analyzed samples.

Sample code	Dental plaque **(n=48)**	Bacterial isolates (n=300)	Dental plaque serotype (n=9)	Bacterial isolates serotype (n=35)	Genes (*cnm/cbm*)
2	Negative	1 positive (*1/15)	Undetected	*e*	Undetected
9	Positive	5 positive (*5/5)	*e*	*e*	Undetected
13	Positive	2 positive (*2/15)	*e*	*e*	Undetected
16	Positive	11 positive (*11/15)	*c*	*c*	Undetected
24	Negative	1 positive (*1/15)	Undetected	*c*	Undetected
26	Positive	5 positive (*5/15)	*c* and *k*	*c* and *k*	Undetected
31	Positive	5 positive (*5/5)	*c*	*c*	Undetected
06 (control)	Positive	5 positive (*5/5)	*c*	*c*	Undetected
13 (control)	Positive	NF (*0/15)	*c*	Undetected	Undetected

* Proportion of isolates positive for *S.mutans* among the analyzed isolates from each individual.

Serotyping of *S. mutans*-positive plaque samples from infarcted patients identified serotypes *c* (samples 16, 26, and 31), *e* (samples 09 and 13), and *k* (sample 26). Similarly, serotyping of bacterial isolates from infarcted patients (n=30) confirmed the presence of these three serotypes (Table 3). In contrast, only serotype *c* was detected in plaque samples (samples 06 and 13) and bacterial isolates (n=5) from the control group (Table 3).

Among *S. mutans*-positive samples from infarcted patients, serotype *c* was observed in 57.1% (4/7), serotype *e* in 42.9% (3/7), and serotype *k* in 14.3% (1/7) (Table 3). In the control group, all *S. mutans*-positive samples (2/2) consisted exclusively of serotype *c* (Table 3). Notably, uncommon serotypes (*e* or *k*) and mixed serotypes (*c* plus *k*) were detected in 57.1% (4/7) and 14.3% (1/7) of *S. mutans*-positive samples from infarcted patients, respectively (Table 3).

PCR amplification for the detection of *cnm* and *cbm* genes in dental plaque samples and bacterial isolates did not yield specific DNA bands for these genes in either the infarcted or control groups (Table 3). Thus, collagen-binding protein genes were absent in all investigated samples.

Pearson's chi-square test demonstrated no significant association between the presence of *S. mutans* (regardless of serotype) and dental caries across the study population, including infarcted patients (p=0.853) and the control group (p=0.787) (Table 4).

**Table 4:** Relationship of *S.mutans* with dental caries in the studied population.

Presence of *S.mutans* (Yes/No)	Association between the presence of *S.mutans* and dental caries				TOTAL	*p*
	**Yes**		**No**			
	**n**	**%**	**n**	**%**		
**With caries in the studied population**						
Yes	7	14.6%	28	58.3%	35	0.885
No	2	4.2%	11	22.9%	13	
**With caries in the infarcted group**						
Yes	6	19.4%	19	61.3%	25	0.853
No	1	3.2%	5	16.1%	6	
**With caries in the control group**						
Yes	1	5.9%	9	52.9%	10	0.787
No	1	5.9%	6	35.3%	7	

Pearson's Chi-square test.

Table 5 summarizes the lipid and glycemic profiles of the study participants. Among infarcted patients, 74.3% exhibited high-density lipoprotein (HDL) cholesterol levels below the desirable range. Analysis of lipid disorders revealed a greater tendency toward myocardial infarction in individuals with low HDL cholesterol levels. Conversely, normal lipid levels conferred protection against myocardial infarction, with a 62.5% association between the variables. Binary logistic regression further indicated that normal cholesterol levels significantly reduced the probability of infarction among non-carriers of *S. mutans*, with a 47.4% lower risk (OR = 0.474; 95% CI = 0.956–0.992).

**Table 5:** Lipid and glycemic profiles of the studied population.

Groups	Normality parameters (mg/dL)				
	GL	TC	HDL-c	LDL-c	TG
	(<100)	(<190)	(>40)	(<130)	(<150)
Infarcted	101.7±32.3	160.8±52.8	34.4±6.4	97.3±48.6	131.7±87.5
*Infarcted with S.mutans*	106.3±51.9	181.4±89.7	36.7±8.3	128.8±80.8	106.4±53.9
Control	89.5±8.8	205.8±34.1	55.0±12.9	127.1±28.7	124.8±66.3
Control with *S.mutans*	93.5±6.4	253±2.8	63.5±23.3	162.1±10.0	135±55.1

GL, glucose. TC, total cholesterol. HDL-c, high-density lipoprotein cholesterol. LDL-c, low-density lipoprotein cholesterol. TG, triglycerides Faludi, et al.^21^ 2017.

## Discussion

In this study, *S. mutans* was detected in 22.6% (7/31) of infarcted patients and 11.8% (2/17) of controls by means of dental plaque and bacterial isolate analysis. These prevalence rates are substantially lower than previously reported values of 95.1% and 100% in oral samples collected prior to cardiovascular surgeries.^5,8,33^ The reduced detection observed here may reflect improved oral hygiene practices among infarcted patients, as plaque samples were obtained post-myocardial infarction following educational interventions highlighting the link between oral and cardiovascular health.

The distribution of *S. mutans* serotypes in infarcted patients revealed serotype *c* as the most prevalent (57.1%, 4/7), followed by *e* (42.9%, 3/7) and *k* (14.3%, 1/7). These findings align with a prior study reporting a 59.0% prevalence of serotype *c* in cardiac patients.^8^ Notably, serotype *f* was absent in our cohort, differing from earlier studies that identified higher frequencies of *e* and *k* in both dental and atheromatous plaques of cardiac patients, while *c* remained predominant in healthy individuals.^7,8^ These results highlight the importance of understanding serotype-specific distribution, as less common serotypes (*e* and *k*) are increasingly associated with cardiovascular diseases.

Regarding serotype diversity, 85.7% (6/7) of *S. mutans*-positive infarcted patients exhibited a single serotype, while one patient (14.3%) carried two serotypes (*c* and *k*). This pattern is consistent with findings from Jordan, the United States, and Spain ^20,34,35^ but diverges from studies in Japan and Thailand, where co-detection of serotypes *c* and *e* was more common.^26^ Interestingly, studies involving patients undergoing cardiovascular surgery have reported a higher prevalence of multiple serotypes,^8^ suggesting that serotype diversity may be linked to disease severity or underlying health conditions.

Additionally, one isolate from a dental plaque sample of a 48-year-old infarcted patient with no comorbidities was confirmed as serotype *k*. Serotype *k*, initially isolated from the blood of individuals with bacteremia, is characterized by a significant reduction in the glucose side chains of the cell wall rhamnose polymer.^36^ *S. mutans* can enter the bloodstream through trauma from dental procedures.^32^ Furthermore, serotype *k* is associated with a lack of glucan-binding protein A (GbpA) expression, resulting in lower sucrose-dependent adhesion and glucan-binding capacity.^26^ It also shows reduced expression of protein antigens, low cellular hydrophobicity, and increased resistance to phagocytosis by polymorphonuclear leukocytes—traits that contribute to systemic virulence.^6,26,32^

Although the clinical relevance of *S. mutans* in systemic diseases has been linked to the Cnm and Cbm proteins,^9,10^ this study did not detect the *cnm* and *cbm* genes in any of the analyzed samples, including those from infarcted patients. Future studies involving samples from various sites, such as cavitated caries lesions and endodontic infections, may provide insights into the distribution and prevalence of *cnm* and *cbm* genes in *S. mutans* strains in Brazil, as strains expressing these proteins are known to have a predilection for collagen-rich tissues.^11,27^

Furthermore, no association was observed between *S. mutans*, regardless of serotype, and dental caries in the studied population. This result may reflect the absence of caries activity assessments in this study, as previous research indicates significantly higher *S. mutans* levels in individuals with active caries.^37^ Additionally, the low prevalence of caries lesions in the population may have influenced the findings.

AMI is often preceded by the rupture or erosion of an atherosclerotic coronary plaque.^38^ Studies have indicated that periodontal and cariogenic bacterial infections, including those caused by *S. mutans*, may contribute to the development and progression of atherosclerosis.^5,8,39^ Low HDL-associated cholesterol is also a recognized risk factor for coronary heart disease.^40^ In this context, our binary logistic regression analysis demonstrated that individuals who did not carry *S. mutans* and presented normal total cholesterol levels had a 47.4% lower probability of experiencing AMI compared to those with altered cholesterol levels and/or presence of *S. mutans* (OR = 0.474; 95% CI = 0.956–0.992).

This study has several limitations. First, the absence of sonication during sample processing represents a methodological constraint, as sonication facilitates biofilm dispersion and the release of microorganisms adhered to tooth surfaces, thereby increasing the sensitivity of microbiological detection.^41^ Without this step, bacterial load and species diversity may have been underestimated, which should be considered when interpreting the results.

Second, no statistical analyses were performed for systemic variables such as fasting blood glucose. Although mean values were higher among infarcted patients than in controls, significance testing was not conducted. Since our main objective was to characterize *Streptococcus mutans*, its serotypes, and collagen-binding genes, the comparative evaluation of metabolic parameters was not prioritized. Future studies should include statistical analyses of these variables to more comprehensively explore associations between systemic conditions, such as hyperglycemia, and cardiovascular risk.^42-43^

Finally, other limitations include the use of plaque samples collected at varying times after infarction and the absence of information on dietary and hygiene habits, factors closely linked to caries development and potentially influencing *S. mutans* prevalence. Additionally, the cross-sectional design, limited sample size, and difficulty recruiting participants under 50 years—given the rarity of AMI in this age group—highlight the need for larger studies to confirm these findings.

## Conclusion

Dental plaque from patients with a history of AMI harbors distinct *S. mutans* serotypes, with a higher frequency of multiple serotype occurrences compared to non-infarcted individuals. Notably, no collagen-binding protein genes (*cnm* or *cbm*) were detected in any *S. mutans* strains analyzed. Furthermore, individuals without *S. mutans* and with normal cholesterol levels demonstrated a lower risk of myocardial infarction.
